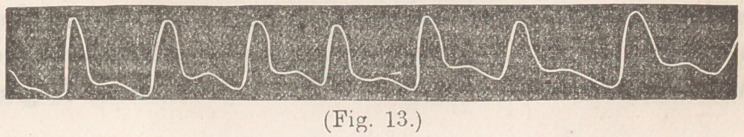# Tracings of the Pulse with Marey’s Sphygmograph

**Published:** 1867-11

**Authors:** H. A. Johnson

**Affiliations:** Prof. Diseases of the Circulatory and Respiratory Organs, in Chicago Medical College


ARTICLE XLVIII.

### TRACINGS OF THE PULSE WITH MAREY'S SPHYGMOGRAPH.

By H. A. JOHNSON, M.D., Prof. Diseases of the Circulatory and Respiratory Organs, in Chicago Medical College.

Read to the Illinois State Medical Society, June, 1867.

(Fig. 1.)

1st. Trace of dicrotic pulse, taken with the sphygmograph from the radial artery of a male patient, *æt.* 35, with typhoid fever, at the end of the third week. Passive congestion of both lungs had just taken place. Treated with quinine in liberal doses, with camphor and morphia. Patient recovered.

(Fig. 2.)

2d. Trace from patient affected with typhoid fever. Male, aged 40; native of Nova Scotia. Taken sick in Nebraska. Intestinal complications not troublesome. Trace taken at the end of the third week. Patient treated with chloride of sodium. Recovered.

(Fig. 3.)

Trace from patient affected with typhoid fever. Male, aged 30. Bowels troublesome; lungs suddenly congested. Trace taken at the end of the third week, at the very commencement of the pulmonary complication. *Treatment.*—Emulsion of turpentine and laudanum, with quinine, camphor, and strychnia. Died.

(Fig. 4.)

From a patient having typhoid fever. Male, aged 24. Came under treatment at the tenth day. Lungs badly congested; great prostration. Trace taken on the tenth day. Died on the twentieth day.

(Fig. 5.)

Pneumonia of right lung. Male, aged 20, Swede. Came under treatment on the fourth day. Trace taken at first visit. Treated with calomel, opium, and ipecac., and afterwards with iodide of potassium, blisters, expectorants, etc. Patient rapidly recovered.

(Fig. 6.)

Pneumonia of right lung, involving nearly the whole of the lung. Male, aged 30. 1st trace (Fig. 6.) taken on sixth day. Treated with cathartics and iodide of potass., followed with

(Fig. 7.)

mixture of scil., ip., sanguinaria, and opium. 2d trace (Fig. 7. taken on the 10th day. Patient recovered.

(Fig. 8.)

Prostration from chronic gastritis. Male, aged 30. Has been sick six months. Has paroxysms of cough, attended with great distress; lungs healthy.

(Fig. 9.)

Rheumatism. Male, aged 40. Had rheumatism first about five years ago. 1st trace (Fig. 9.) taken on coming under

(Fig. 10.)

treatment. Put upon colchicum, in full doses. 2d trace (Fig. 10.) after six days' treatment. Left cured on the 10th day.

(Fig. 11.)

Rheumatism, not severe. Male, aged 43.

(Fig. 12.)

Contraction of aortic opening. Female, aged 8 years.

(Fig. 13.)

Functional disturbance of heart's action. Male, aged 50, German. Has had trouble with heart for a long time; has also been treated for disease of the kidneys; heart sounds normal in quality, but irregular in rythm and intensity. The trace shows the ordinary movements of the organ. Not much inconvenience is experienced. Did not know that anything was the matter with his heart till told so by a physician. Urine loaded with the urate of ammonia; otherwise normal.

## Figures and Tables

**Fig. 1. f1:**
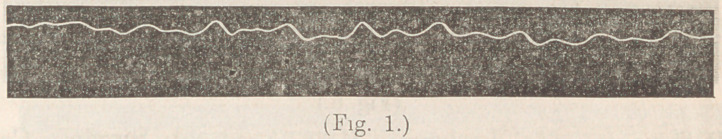


**Fig. 2. f2:**
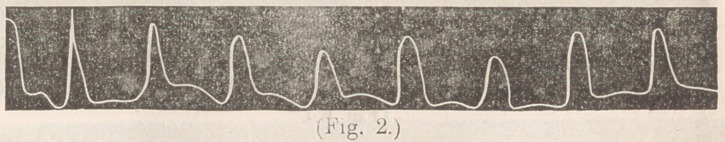


**Fig. 3. f3:**
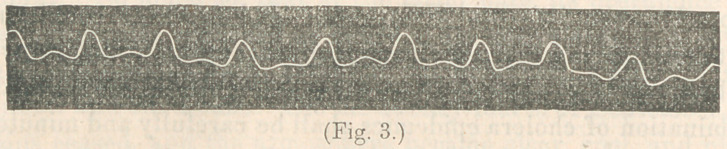


**Fig. 4. f4:**
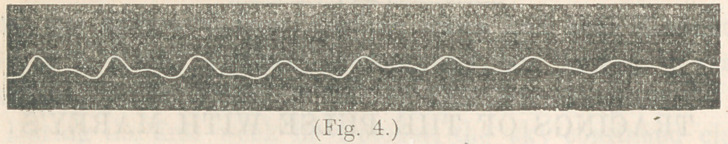


**Fig. 5. f5:**
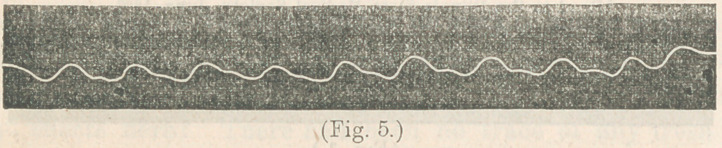


**Fig. 6. f6:**
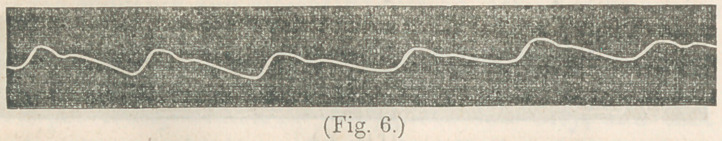


**Fig. 7. f7:**
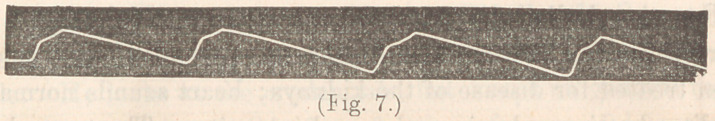


**Fig. 8. f8:**
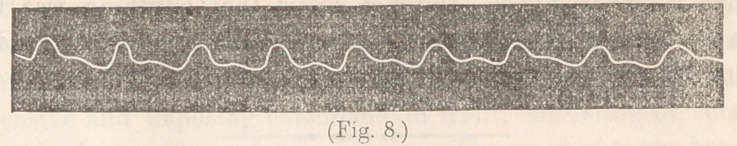


**Fig. 9. f9:**
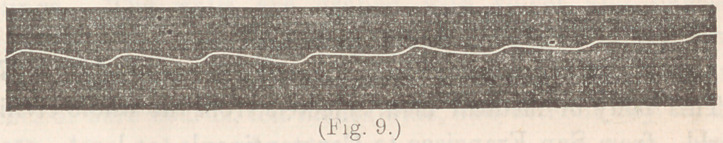


**Fig. 10. f10:**
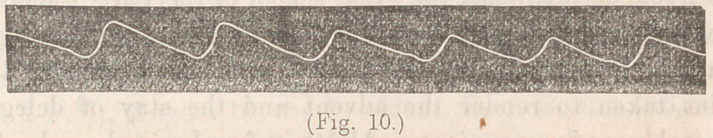


**Fig. 11. f11:**
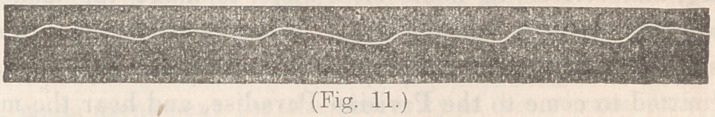


**Fig. 12. f12:**
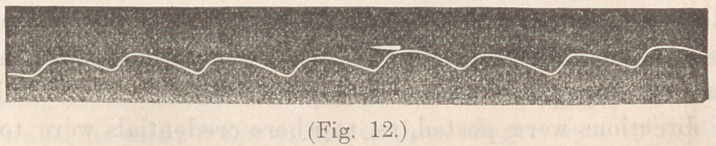


**Fig. 13. f13:**